# Mechanisms of Cardiovascular Protection Associated with Intermittent Hypobaric Hypoxia Exposure in a Rat Model: Role of Oxidative Stress

**DOI:** 10.3390/ijms19020366

**Published:** 2018-01-26

**Authors:** Miguel Aguilar, Alejandro González-Candia, Jorge Rodríguez, Catalina Carrasco-Pozo, Daniel Cañas, Claudio García-Herrera, Emilio A. Herrera, Rodrigo L. Castillo

**Affiliations:** 1 Programa de Fisiopatología, Instituto de Ciencias Biomédicas, Facultad de Medicina, Universidad de Chile, Santiago 7500922, Chile; miguel23.aguilar@gmail.com (M.A.); alejjobq@gmail.com (A.G.-C.); jrodborg@hotmail.com (J.R.); 2 Departamento de Kinesiología, Facultad de Medicina, Universidad de Chile, Santiago 8380453, Chile; 3 Discovery Biology, Griffith Institute for Drug Discovery, Griffith University, Brisbane, QLD 4222, Australia; c.carrascopozo@griffith.edu.au; 4 Departamento de Nutrición, Facultad de Medicina, Universidad de Chile, Santiago 8380453, Chile; 5 Departamento de Ingeniería Mecánica, Facultad de Ingeniería, Universidad de Santiago de Chile, Santiago 9170125, Chile; daniel.canas@usach.cl (D.C.); claudio.garcia@usach.cl (C.G.-H.); 6 International Center for Andean Studies, Universidad de Chile, Putre, Chile

**Keywords:** intermittent hypobaric hypoxia, oxidative stress, heart, vascular response

## Abstract

More than 140 million people live and works (in a chronic or intermittent form) above 2500 m worldwide and 35 million live in the Andean Mountains. Furthermore, in Chile, it is estimated that 55,000 persons work in high altitude shifts, where stays at lowlands and interspersed with working stays at highlands. Acute exposure to high altitude has been shown to induce oxidative stress in healthy human lowlanders, due to an increase in free radical formation and a decrease in antioxidant capacity. However, in animal models, intermittent hypoxia (IH) induce preconditioning, like responses and cardioprotection. Here, we aimed to describe in a rat model the responses on cardiac and vascular function to 4 cycles of intermittent hypobaric hypoxia (IHH). Twelve adult Wistar rats were randomly divided into two equal groups, a four-cycle of IHH, and a normobaric hypoxic control. Intermittent hypoxia was induced in a hypobaric chamber in four continuous cycles (1 cycle = 4 days hypoxia + 4 days normoxia), reaching a barometric pressure equivalent to 4600 m of altitude (428 Torr). At the end of the first and fourth cycle, cardiac structural, and functional variables were determined by echocardiography. Thereafter, ex vivo vascular function and biomechanical properties were determined in femoral arteries by wire myography. We further measured cardiac oxidative stress biomarkers (4-Hydroxy-nonenal, HNE; nytrotirosine, NT), reactive oxygen species (ROS) sources (NADPH and mitochondrial), and antioxidant enzymes activity (catalase, CAT; glutathione peroxidase, GPx, and superoxide dismutase, SOD). Our results show a higher ejection and shortening fraction of the left ventricle function by the end of the 4th cycle. Further, femoral vessels showed an improvement of vasodilator capacity and diminished stiffening. Cardiac tissue presented a higher expression of antioxidant enzymes and mitochondrial ROS formation in IHH, as compared with normobaric hypoxic controls. IHH exposure determines a preconditioning effect on the heart and femoral artery, both at structural and functional levels, associated with the induction of antioxidant defence mechanisms. However, mitochondrial ROS generation was increased in cardiac tissue. These findings suggest that initial states of IHH are beneficial for cardiovascular function and protection.

## 1. Introduction

Several studies have reported that intermittent hypoxic (IH) training can provide evident measurable protection in some disease states or enable improvements in selected sports related performances [1,2,3]. The protective effects of IH can be explained by the activation and propagation of homeostatic or adaptive responses elicited by the IH stimulus, usually through a process that has been generally termed as preconditioning. Thus, short exposures to mild IH episodes can afford protection to specific cells, tissues, or organs against more severe hypoxia and ischemia [4].

Animals subjected to various paradigms of acute IH become more resistant to the lethal injury induced by subsequent exposures to severe hypoxic insults [5,6,7]. For instance, when compared to controls, mice treated with brief episodes of low frequency IH (8% O_2_ × 10 min/21% O_2_ × 10 min, six cycles) showed attenuated cellular and tissue injury to crucial organs, such as the lung and the brain, relative to extended hypoxia. Furthermore, myocardia from mice treated with a similar IH pattern (6% O_2_ × 6 min/21% O_2_ × 6 min, five cycles) or from rats treated with a higher frequency but short duration IH (10% O_2_ × 40 s/21% O_2_ × 20 s, for 4 h) were protected against ischemia-induced infarction [8,9]. Such IH induced cardioprotection seemed to be dependent on the activation of similar pathways to those described in models of cardiac ischemic preconditioning, in which sufficient expression and activity of hypoxia inducible factor 1 α (HIF-1α) is required [9,10]. Also, intermittent hypoxia may protect the heart against ischemia reperfusion (IR) injury by diminishing ischemia induced contractile dysfunction [11], endothelial dysfunction, arrhythmias, and cell death [12]. This protection has been ascribed to higher myocardial vascularity, coronary blood flow, cardiomyoglobin and expression of antioxidant enzymes induced by IH [13]. In addition, IH appears to provide a therapeutic effect on permanent coronary artery ligation induced myocardial infarction by reducing the infarct size, myocardial fibrosis, and apoptosis [14]. Further, it has been shown that four cycles of hypobaric hypoxia are able to improve cardiac infarction recovery and this has been associated with an enhanced antioxidant enzymes expression [15].

However, the mechanism that support the precondition effects of acute IH on myocardial and vascular function have not been well characterized. The aim of this work is to describe the responses on cardiac and vascular function, structure and biomechanical properties after four cycles of intermittent hypobaric hypoxia (IHH) and the association with pro oxidant markers and ROS sources in a well-established rat model [15,16]. 

## 2. Results

### 2.1. Animals Weights

The weights of the control rats were measured at the entrance of the first cycle (NN1: 279 ± 13 vs. IHH1: 285 ± 6 g) and at the end of the fourth cycle (Table 1), with no differences between groups. Further, some abdominal and thoracic organs were weighed at post mortem at the end of the 4th cycle. The spleen weight was increased in the IHH group. In contrast, all the rest of the organs presented similar weights between groups (Table 1).

### 2.2. In Vivo Cardiac Morphometry & Function

The left ventricular diastolic diameters (LVDD) showed a significant decrease in the IHH rats at the end of the fourth cycle relative to controls (*p* = 0.035). In addition, the systolic diameter of the left ventricle (LVSD) decreased significantly in the first and fourth cycles in IHH animals (*p* = 0.029). However, the diastolic thickness of the interventricular septum (IVSD), Left ventricle free wall (LVWD), left atrium diameter (LADD), and aortic diameter (ADD) were similar between groups (Table 2). Further, the systolic function measured by Doppler (Vmax, Vmed, GPmax & GPmed, by RVA-716 visualSonics Toronto, ON, Canada) was increased in IHH1 and IHH4 relative to normoxic groups. In contrast, E-wave was similar between groups (Table 2). 

**Figure 1:**
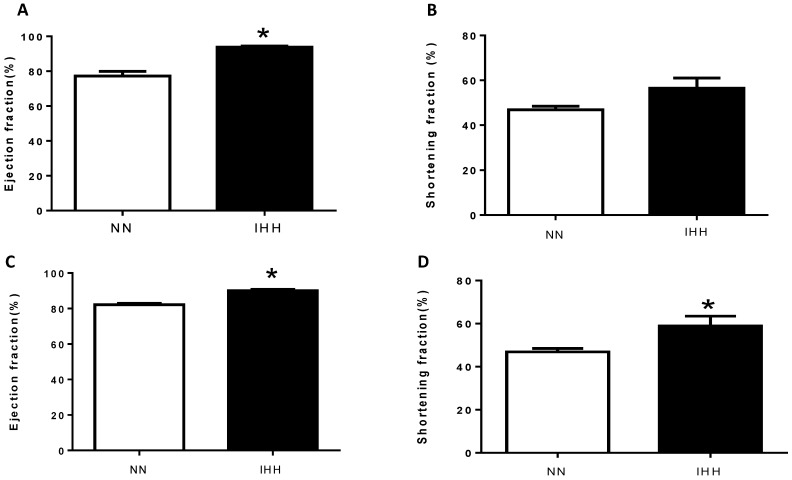
Echochardiographic heart function. The left heart function was evaluated through the ejection fraction (EF%, (**A**,**C**)) and shortening fraction (SF%, (**B**,**D**)). The first measurement was performed following the first cycle of intermittent hypobaric hypoxia (**A**,**B**) and the second one was performed following the fourth intermittent hypobaric hypoxia (IHH) cycle (**C**,**D**). Data are expressed in mean ± SEM. Significant differences (* *p* ≤ 0.05) vs. Normobaric normoxia.

The ejection fraction (EF%) of the IHH group showed significant increases of 22.85% at the end of the first cycle and of 7.88% at the end of the fourth cycle when compared to controls (*p* = 0.041; *p* = 0.033, respectively). In addition, the shortening fraction (SF%) in the first cycle showed no difference, whereas at the end of the 4th cycle, it presented a significant increase of 9.56% when compared to the control group (*p* = 0.027) (Figure 1). Finally, heart rate was similar between groups at the end of the first cycle, but there was a marked increase in the IHH group by the end of the fourth cycle (Table 2).

### 2.3. Ex Vivo Femoral Vascular Function Active Response

**Figure 2:**
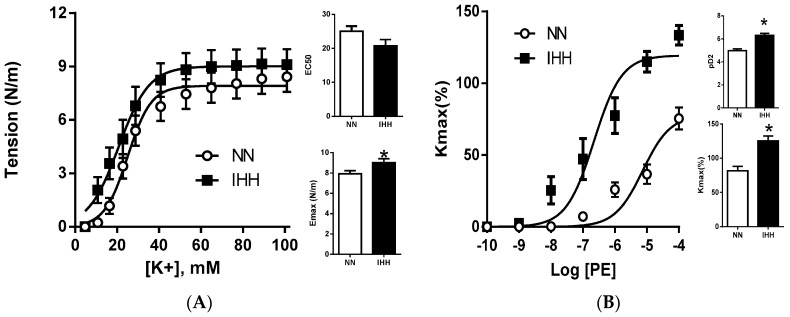
Femoral vascular function. Vasoconstriction was induced by KCl (**A**) and phenylephrine (PE, (**B**)). Maximum response is expressed in Emax and Kmax, sensibility is expressed by EC50 and pD2, respectively (inserted histograms). Data are expressed in mean ± SEM. Significant differences (* *p* ≤ 0.05) vs. Normobaric normoxia.

K^+^ induced vasoconstriction showed a higher sensitivity in the IHH group (pD2: NN, 25.05 ± 1.49 vs. IHH, 20.76 ± 1.83 mM) (*p* = 0.025), with similar contractile capacity (Emax: NN, 7.91 ± 0.30 vs. IHH, 9.01 ± 0.38 N/m). In addition, phenylephrine induced vasoconstriction showed both higher sensitivity (pD2: NN, 4.97 ± 0.14 vs. IHH, 6.30 ± 0.17) (*p* = 0.042) and maximal effect (Kmax: NN, 81.46 ± 6.66 vs. IHH, 125.30 ± 7.16%) in the IHH group (*p* = 0.032) (Figure 2).

### 2.4. Ex Vivo Femoral Vascular Function—Passive Response

**Figure 3:**
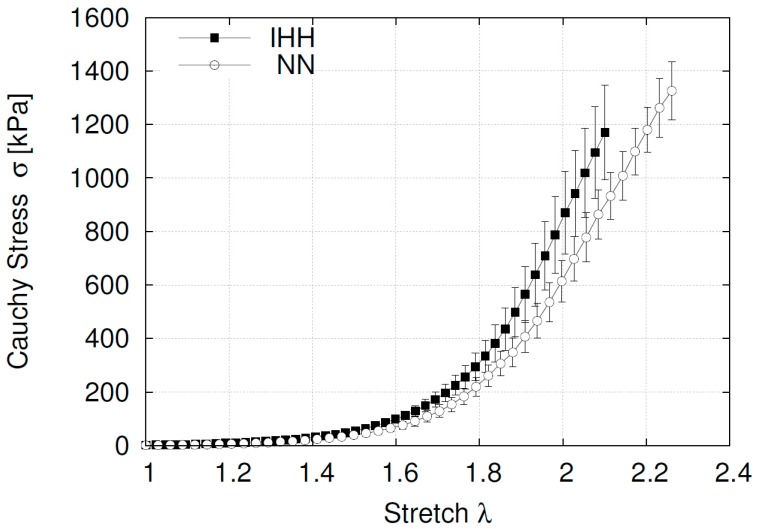
Stress-stretch curve for femoral arteries. Data are expressed in mean ± SEM for the curve progression.

The stress strain curves of the femoral arteries showed similar initial slopes between groups (NN, 39.04 ± 8.45 vs. IHH, 40.87 ± 5.77 kPa, with no significant differences in the experimental curves elbow (NN: 1.79 ± 0.05 vs. IHH: 1.74 ± 0.01). However, the end of the stretching process enhanced the slope enhanced in the IHH group relative to the NN animals (NN, 2415 ± 229 vs. IHH, 3427 ± 309 kPa) (*p* = 0.031), indicating an increased stiffness in the hypoxic animals (Figure 3).

### 2.5. Cardiac Antioxidant Capacity & Anion Sources

**Figure 4:**
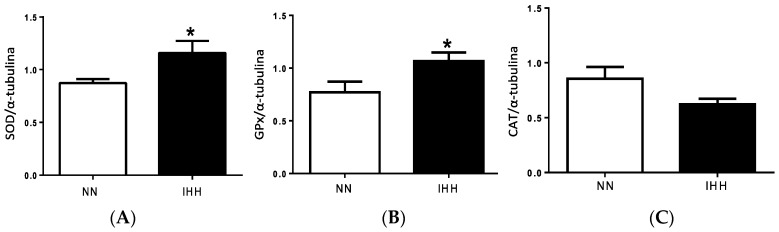

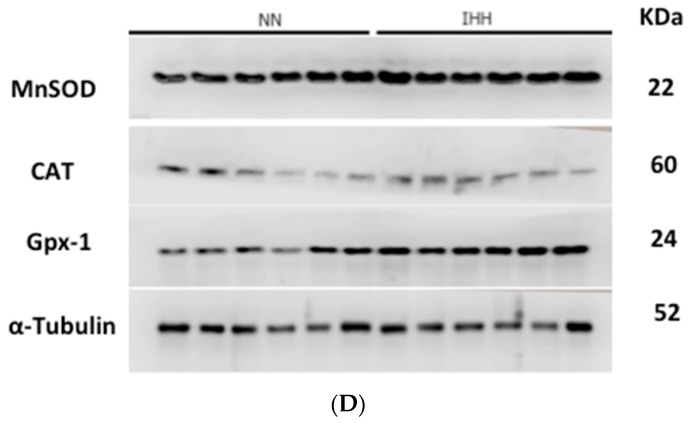
Antioxidant enzymes expression. Superoxide dismutase (SOD) (**A**); Glutathione peroxidase (GPx) (**B**); Catalase (CAT) (**C**) protein expression; and densitometric assay (**D**). Data are expressed in mean ± SEM. Significant differences (* *p* ≤ 0.05) vs. Normobaric normoxia.

**Figure 5:**
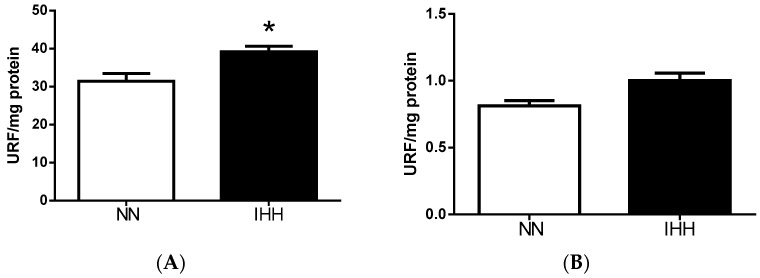
Reactive oxygen species generation. Reactive oxygen species (ROS) production was measured through DHE oxidation (mitochondrial source, (**A**)) and nicotinadine adenine dinucleotide phosphate oxidase (NADPH oxidase, (**B**)) in heart of rats. Data are expressed in mean ± SEM. Significant differences (* *p* ≤ 0.05) vs. Normobaric normoxia.

Antioxidant enzymes SOD and GSH PX presented a significant increase of 28.55% and 29.44%, respectively, in the IHH group relative to the NN group (*p* = 0.022; *p* = 0.037). However, CAT levels were similar between both groups (Figure 4). NADPH oxidase production of superoxide anion (•O_2_^−^) was similar between groups. In contrast, the mitochondrial generation of •O_2_^−^ was higher in IHH when compared to NN animals (Figure 5 and Figure S1) (*p* = 0.030). 

### 2.6. Cardiac Biomarker of Hypoxia and Oxidative Stress

**Figure 6:**
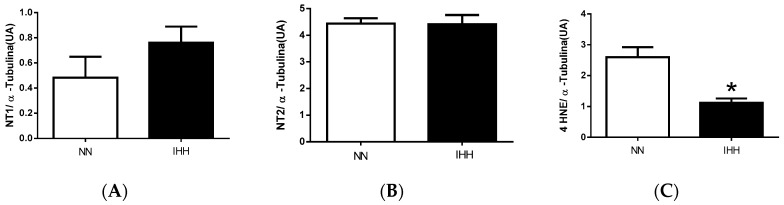

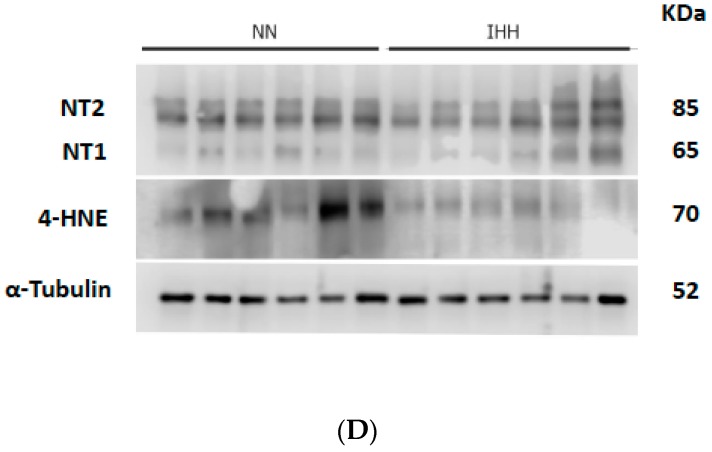
Oxidative stress markers. Levels of nitrotyrosine-1 (NT1, (**A**)); nitrotyrosine-2 (NT2, (**B**)); and 4 Hydroxynonenal (4 HNE, (**C**)); and densitometric assay (**D**). Data are expressed in mean ± SEM. Significant differences (* *p* ≤ 0.05) vs. Normobaric normoxia.

HNE levels were lower in the IHH group relative to NN group; however, NT expression was similar between both groups (Figure 6 and Figure S1). 

## 3. Discussion

In the present study, we demonstrate that IHH exposure determine a preconditioning effect on the heart and femoral artery, both at structural and functional levels, associated with the induction of antioxidant defence mechanisms. These findings indicate that initial states of IHH are beneficial for cardiovascular function and protection.

Few studies have evaluated the effects of intermittent hypobaric hypoxia, in which the apparent capacity of cardiac protection stands out dependent on a preconditioning-like effect [15]. It seems that this preconditioning phenomenon may only be induced by few repetitive exposures to hypobaria [17]. In most cases, this seems to be true, since in long periods the results are not consistent, and are even contradictory. However, the difference in results could be due to the diversity of simulated altitudes and/or exposure time [18]. 

Currently, our findings in cardiac systolic function show a greater ejection fraction, both at the end of the first cycle and at the end of the fourth cycle in the HIA rats. This adaptation apparently starts early, affecting even from the first exposure cycle, and the prolongation of the stimulus increased the shortening fraction. In the literature, we found similar data in acute hypobaric hypoxia during exercise in humans, when compared in the same subjects at rest [19].

With respect to the occurrence of oxidative stress, a pro-oxidant state is known to be associated with various forms of cardiac pathology [20,21]. In these conditions, cardiac function may be impaired due to increased oxidative stress and the formation of substances, such as peroxynitrite, which inhibits SERCA protein, altering cytosolic calcium kinetics [22,23]. Therefore, in a decreased oxidative stress intracellular environment, there would be more calcium available in the cytoplasm that binds to troponin C, finding a greater number of actin-myosin binding sites, improving contractility of the cardiomyocyte. This could be the cause of the EF% increase in acute exposure. On the other hand, the immediate acute responses does not involve an increase in the heart rate, but it does increase when the stimulus is prolonged at the end of the 4th cycle. Previous studies, such as those carried out by Boos et al. [19], are consistent with our results and note that this effect is observed in all forms of acute intermittent hypobaric hypoxia. A possible mechanism inducing this effect is the increase in the sympathetic tone described within the acclimatization processes to hypobaric hypoxia [24,25], which would generate both an increase in chrono-tropism and inotropism [26]. Within these effects, β-adrenergic agonism increases the second messenger cyclic adenosine monophosphate (cAMP), which binds to and activates a cAMP-dependent protein kinase (PKA). PKA has different cell substrates at the myocardial level as targets for phosphorylation: phosphorylation of L type calcium channels of the cell membrane and ryanodine receptors of the sarcoplasmic reticulum, provoking an increase in intracellular free Ca^2+^ concentration [27].

When analyzing the cardiac structure by echocardiography at the end of the 4th cycle, there is a decrease in the internal diameter of the left ventricle in diastole. This finding is striking because the contractile capacity of the heart would not be apparently related to Starling’s law [28], and the increase in preload, but rather to contractility of the cardiomyocyte itself. Therefore, we believe that systolic function improvement in rats exposed to HIA is associated with an increase in myocardial contractility [29]. This cardioprotective effect could be due to the increase of the antioxidant capacity initially, and later to the sympathetic stimulation.

Moreover, vasoconstrictor function in the carotid and femoral arteries are also affected, with effects similar to those described by increased smooth muscle area in a chronically hypoxic rat model [30]. While this is consistent with the literature that the increased stimulation of catecholamines and serotonin in hypoxia generates a mitogenic effect, it is interesting to note that this effect is found even in short times of exposure to hypoxia. This is probably an adaptive change, which generates an increase in peripheral vascular resistance [31].

Hypoxia increases the production of ROS associated with various sources, mitochondria, xanthine oxidases, NADPH oxidases [32,33], an increase that is proportional to height [34]. In spite of this, we have observed that there is a decrease in oxidative stress associated with an increase in enzymatic antioxidant capacity in intermittent hypobaria [15]. Accordingly, our findings demonstrate an increase in the expression of SOD and GSH-Px, data that are in agreement with previous studies [34]. It is likely that this effect is associated with the erythroid 2 nuclear factor (Nrf2) [35] actions, which stimulates the transcription of antioxidant enzymes, thus increasing the antioxidant capacity (enzymatic and non-enzymatic) in a compensatory way to a ROS increase [36]. 

Indeed, HIF expression is known to be the major regulatory factor in response to hypoxia, which regulates several cellular and molecular changes. For example, HIF-1a is necessary for the early window of ischemic preconditioning; ameliorate ROS damage and induce antioxidant enzymes expression [9,15,37]. The higher expression of HIF-1a at IHH (4 cycles) has been previously described by our group in this animal model [15]. Indeed, inhibition of HIF degradation with PHD inhibitors, as well as remote preconditioning (in part through HIF), might develop into novel clinical interventions in organ protection, such as myocardial infarction and in organ transplantation [38].

These changes described are in according to a pre-conditioning process and a decrease in myocardial and vascular damage. 

Andean mountain medicine has repeatedly shown that populations that are chronically exposed to altitude have low incidence of hypertension, atherosclerosis and myocardial infarction [39]. Is possible that these characteristics depend on chronic hypoxia exposure, racial or nutritional factors have not yet been elucidated [40]. Similarly, it has been recognized that long-term high altitude hypoxia exposure protects the heart against hypoxic injury, inducing a reduction of infarct size during acute ischemia [29,41]. Further, the cardiac function recovery after a period of ischemia was improved in IHH initial process [15]. The relaxing effects of hypoxia on arterial smooth muscle cells may be proposed as contributors to this protection, as the vasodilatation tends to counteract polycythemia-induced blood viscosity [42]. Overall, the initial cardiovascular effects of altitude exposure involve an acute response that is associated with increased heart rate, blood pressure, cardiac output, and contractility [43]. Over time, during chronic hypoxia cardiac output decreases at levels lower than pre-exposure, accompanied by a decrease in sympathetic activity secondary to cardiac β-adrenergic receptor desensitization [44]. In miners exposed to CIH for 31 months, blood pressure initially increased followed by a reduction, but remained slightly elevated whencompared to blood pressure measured at sea level; also a reduction in pulse and a slight dilation of the right ventricle were observed [45,46]. A study performed on Chilean soldiers exposed to CIH for more than 12 years revealed an increase in the amount of triglycerides and a reduction in LDL cholesterol [47]. These findings are in agreement with a protective effect of intermittent hypoxic exposure, for example, as an effective alternative to chronic altitude residence for increasing resting ventilation and reducing the incidence and severity of acute mountain sickness [48]. However, other metabolic effects on insulin sensitivity or the antiatherogenic effect of CIH, only described in animal models, has been poorly studied in a mechanistic way [49]. Additional involved mechanisms require further studies due to the impact on cardiovascular risk [50].

Stem cells transplanted to the ischemic myocardium usually encounter massive cell death within a few days after transplantation, and hypoxic preconditioning is currently used as a strategy to prepare stem cells for increased survival and engraftment in the heart. For instance, in vitro studies show that hypoxic preconditioning causes a reduction of proapoptotic elements (cytochrome c and cleaved caspase-3) and the preservation of anti-apoptotic components of the mitochondria (Bcl-2, Bcl-XL and p-Bad) [51]. Further, hypoxic preconditioning enhances the benefit of cardiac progenitor cell therapy for treatment of myocardial infarction [52].

Some reports indicate that exposure to high altitude hypobaric hypoxia causes oxidative cellular damage. This cellular oxidative stress appears directly related to the altitude level and an increased production of ROS seems to be responsible for these effects [46,53]. Concomitantly, oxygen enrichment of room air is increasingly being used in work stations at high altitude [54], and since the production of ROS is favoured at higher oxygen supply [55], oxidative stress may be an even more important factor in high altitude exposed workers. In this context, exposure to high altitude reduces activity and expression levels of antioxidant enzymes; consequently, the disruption in the efficiency of the antioxidant systems due to the increase in ROS production by hypobaric hypoxia leads to oxidative damage of macromolecules [56,57]. The main cause of oxidative stress is the lower availability of O_2_ to be reduced to H_2_O by the enzyme cytochrome oxidase in the mitochondrial respiratory chain. However, the effect on cardiovascular function of the redox imbalance has not been clinically well characterized. 

In this study, using a rat model, we found that femoral arteries from IHH vessels have a diminished stiffened response, which is associated to an increase in the Cauchy stress slope post-transition zone (elbow). On the contrary, no changes at the beginning and little variations of the elbow in the transition zone were found between groups. In this context, studies have related the changes in the biomechanical response at the end of the curve to the action of the collagen fibers and orientation, which are responsible for the structural strength at higher loads. On the other hand, elastin is one of the main contributors to the first part of the response [58]. Compelling evidence in rats, show that repetitive cycles of hypoxic exposure are accompanied with new collagen depositions in CIH [59], leading to a stiffened response of the vessels. The latter suggests that some kind of deposition on the tunica might be happening. However, according to our results, the first stage of the curve represents physiological intraluminal pressures, given that the elbows of the stress-stretch curves are near 280 mmHg, with no differences between the normoxic and the hypoxic groups. Therefore, considering that vessels working under a physiological range of pressure could be located around the transition zone of the stress-stretch curve [58] for healthy subjects, the increased arterial stiffness might seem unlikely to be clinically meaningful in this model. However, this also might indicate that longer expositions to IHH can trigger vascular dysfunction. 

## 4. Materials and Methods

All animal care, maintenance, and procedures were approved by the Bioethics Committee of the Faculty of Medicine, University of Chile (16 August 2016) (number of protocol, CBA 0865 FMUCH) and was registered on the Chile as CBA-0865, Santiago, Chile. All of the procedures were carried out in accordance to Guidelines for the Care and Use of Laboratory Animals.

### 4.1. Animals

Twelve male adult Wistar rats (8 weeks of age, 280 g weight were randomly divided into two equal groups: one group maintained in normobaric normoxia (Control, NN) in the hypobaric chamber, while the other was expose to hypobaric hypoxia in the same hypobaric chamber, in intermittent shifts equivalent to 4600 m (428 Torr). The shifts consistent in four cycles (intermittent hypobaric hypoxia, IHH), where each cycle consisted in 4 days in hypobaric hypoxia and 4 days in normobaric normoxia. Standard polycarbonate cages were used, with two rats in each one, environmental temperature of 22–25 °C, humidity of 45–55%, 12 h/12 h light/darkness, standard commercial diet, and water *ad libitum* [15]. 

### 4.2. Echocardiography

The echocardiographic examinations (Sonosite 180 Plus and a 10 MHz linear transducer) were performed by the end of the first cycle and fourth cycles in IHH groups, at the equivalent times in the control normobaric hypoxic group. Ultrasound imagery was performed under a combination of ketamine:xylazine anesthesia (80 mg/kg:10 mg/kg, IP). To assess cardiac morphology and function, we determined heart rate (HR), diastolic (DDLV), and systolic (SDLV) diameters of the left ventricle, thickness of the interventricular septum (IVS) and left ventricle free wall (LVW), aortic diameter, ejection fraction (EF) and shortening fraction (%SF) [60]. 

### 4.3. Tissue Collection

At the end of the 4th cycle and after the echocardiographic examination, all of the animals were euthanized with and anesthetic overdose (Sodium Thiopentone 150 mg∙kg^-1^ IP). Once death was confirmed, the hearts and femoral arteries were excised, weighed, and stored for ex vivo analyses (wire myography), molecular biology, and histological determinations.

### 4.4. Vascular Function Active Response

Femoral vascular function was evaluated by wire myography. Immediately after extraction of both femoral arteries, 2 mm segments were mounted in a wire myograph (model 620M; Danish Myo Technology A/S, Aarhus, Denmark), immersed in Krebs solution with a continuous contribution of 5% CO_2_ and 95% O_2_ at 37 °C [61]. 

After an equilibration period of 15 min, the femoral rings were stretched to achieve a physiological transmural pressure, simulating the in vivo condition. Subsequently, the Krebs solution was maintained for 15–20 min, after which the contractile capacity was determined with incremental doses of K^+^ (4.72–125 mM). In addition, vasoconstrictor function was evaluated using cumulative concentration-response curves (CCRC) to phenylephrine (10^−10^–10^−3^) [61,62].

### 4.5. Vascular Function Passive Response

A different set of femoral arteries segments of 2 mm were mounted in the wire myograph, maintained at 37 °C and were kept in a Ca^2+^ free Krebs buffer with constant bubbling (5% CO_2_, 95% O_2_). The passive response assessment consists in subjecting the vessel segment to a continuous radial elongation until rupture. The initial length was described by:

∆_o_ = π/2 × [d − (Ф + e_o_)] (1)

Using Equation (1); the stretch was defined, considering the semiperimeter of the vessel and the separation of the hooks, as:

λ = 1 + 2 × [ (∆ − ∆o)/(πd)](2)

Finally, the Cauchy stress expression was determined by:

σ = [F/(2a_o_e_o_)] × λ (3)

where the variables used were the initial thickness (e_o_), width (a_o_), the diameter of the hooks (Ф), the mean diameter of the vessel (d), the load (F) and the displacement of the hooks (∆). For the comparison of between vessels, three parameters were summarized to represent the passive mechanical response; the magnitude of the slopes at the beginning and end of the experimental curve, and the elbow of the stress-stretch curve, as described in detail elsewhere [63].

### 4.6. Biomarker of Hypoxia and Oxidative Stress

After euthanasia, cardiac tissue was frozen in liquid nitrogen and stored at −80 °C until use. After tissue homogenization, Western blot (WB) analyses were performed for the protein expression of 4 HNE; specific antibody ab46545 (Abcam Laboratories, Cambridge, UK), Nitrotyrosine (NT, anti-NT 05-233, Millipore, Billerica, MA, USA) as described previously [16,62].

### 4.7. Cardiac Antioxidant Enzymes

Expression of manganese dismutase superoxide (Mn SOD), catalase (CAT) and glutathione peroxidase (GSH Px1) proteins were measured by Western Blot using specific antibodies (antiMn SOD, Millipore), 06-984; anti-Catalase, Abcam Laboratories, ab1877 and antiGSH PX1, Abcam Laboratories, ab22604), as described previously [62].

### 4.8. Reactive Oxygen Species Sources

We determined the generation of superoxide anion (O_2_•−) through the oxidation of 10 μM DHE (470 Ex/590 Em) after incubation for 30 min a 37 °C with the homogenized tissue (20–50 μg protein). In addition, the activity of NADPH oxidase was determined, as described previously [64].

### 4.9. Statistical Analyses

Values were expressed as average and arithmetic mean (interquartile range) ± standard error of the mean (SEM), according to the distribution of variables.

The vascular response to potassium was analyzed using the Boltzmann sigmoidal analysis. Further, the maximal effective tension (E_max_) and the half maximal effective concentration (EC_50_) were determined. All the other CCRCs were analyzed using an agonist response best fit equation, where the maximal vasomotor response was expressed as the percentage of the submaximal contraction induced by K^+^max (64 mM). The sensitivity was expressed as pD2 (−logEC_50_), as published elsewhere [62]. 

The analysis of the passive responses were based on the magnitude of the slope at the beginning (E1) and at the end (E2) of the stress-stretch curve, the stress at the elbow of the stress-stretch curve (σ2) and the stretch and stress at the breaking point (λR, σR), as described previously [63].

Ratios and percentages were arcsine-transformed prior to statistical analysis. All of the results were compared statistically by an unpaired t test. Significant differences were accepted when *p* ≤ 0.05 (Prism 5.0; GraphPad Software, California, CA, USA).

## 5. Conclusions

Intermittent hypobaric hypoxia exposure determines a preconditioning effect on the heart and femoral artery, both at structural and functional levels, which are associated with the induction of antioxidant defence mechanisms. Therefore, using this model in future studies, we can define pharmacological targets in the treatment of cardiovascular disorders induced by IHH. 

The hypoxia in the case of our experimental design is secondary to hypobaria (high altitude and falling PO_2_ values). This mechanism of hypoxia is associated with a clinical condition that validates our model, such as the exposure of the human population in intermittent form (IH). As mentioned in the previous answer, “normobaric” controls cannot be applied in our model, since it corresponds to the effects of OSA. In addition, in the case of O_2_ and SaO_2_ levels in animals, this same model has already estimated these values in another older published paper and the effects have been widely discussed in them [25,33]. We use this strategy of hypobaric hypoxia as an effect of preconditioning of cardiac function.

## Figures and Tables

**Table 1:** Body and organs weight.

Organs Weight	NN	% NN	IHH	% IHH
Body weight (g)	445 ± 15	-	422 ± 13	-
Heart (g)	1.582 ± 0.071	0.355 ± 0.016	1.521 ± 0.049	0.359 ± 0.011
Lung (g)	2.631 ± 0.215	0.590 ± 0.048	2.021 ± 0.294	0.477 ± 0.069
Spleen (g)	1.115 ± 0.062	0.250 ± 0.013	1.347 ± 0.082 *	0.318 ± 0.019
Liver (g)	12.483 ± 0.886	2.803 ± 0.199	10.968 ± 0.883	2.593 ± 0.209
Left kidney (g)	1.317 ± 0.051	0.295 ± 0.011	1.402 ± 0.152	0.314 ± 0.036
Right kidney (g)	1.369 ± 0.047	0.307 ± 0.010	1.329 ± 0.074	0.331 ± 0.017

Measurements of wet weights of organs in grams (g) at the end of the fourth cycle (post-mortem), and percentage (%) measurement according to final body weight. Groups are NN: Normobaric Normoxia, IHH: Intermittent Hypobaric Hypoxia. Data are expressed in mean ± standard error of the mean (SEM). Significant differences (* *p* ≤ 0.05) vs. Normobaric normoxia.

**Table 2:** Echocardiographic variables in first and fourth cycle.

Cardiac Parameters	NN1	IHH1	NN4	IHH4
LVDD (mm)	7.139 ± 0.426	6.498 ± 0.374 *	7.525 ± 0.166	5.957 ± 0.398 *
LVSD (mm)	4.131 ± 0.334	2.965 ± 0.305 *	3.987 ± 0.110	2.642 ± 0.396 *
IVSD (mm)	1.571 ± 0.106	1.600 ± 0.108	1.762 ± 0.147	2.000 ± 0.121
LVWD (mm)	2.501 ± 0.291	3.151 ± 0.245	2.887 ± 0.161	3.014 ± 0.192
LADD (mm)	3.922 ± 0.135	3.527 ± 0.310	4.482 ± 0.110	3.857 ± 0.03
ADD (mm)	3.382 ± 0.247	3.015 ± 0.154	2.907 ± 0.165	2.437 ± 0.247
Vmax (cm/s)	83.41 ± 4.21	128.40 ± 4.71 *	69.58 ± 2.66	139.05 ± 4.67 *
Vmed (cm/s)	47.05 ± 3.46	72.84 ± 4.84 *	50.97 ± 2.14	77.07 ± 4.54 *
GPmax (mmHg)	2.832 ± 0.273	6.615 ± 0.511 *	1.966 ± 0.144	6.941 ± 1.059 *
GPmed (mmHg)	0.918 ± 0.131	2.227 ± 0.303 *	1.467 ± 0.236	2.437 ± 0.247 *
E-Wave (cm/s)	80.72 ± 7.76	56.93 ± 5.42	83.47 ± 6.62	86.47 ± 3.77
HR (bpm)	242 ± 19	243 ± 8	220 ± 11	261 ± 15 *

Measurement of left ventricle diastolic diameter (LVDD), left ventricle systolic diameter (LVSD), interventricular septum during diastole (IVSD), left ventricular free wall in diastole (LVWD), left atrium diameter (LADD), and aortic diameter (ADD), all expressed in millimeters (mm); peak velocity (Vmax), mean velocity (Vmed), and E wave (E-Wave) measured in cm/s; peak systolic ejection gradient (GPmax), and mean systolic ejection gradient (GPmean) measured in mmHg; and heart rate measured in beats per minute (bpm), at the end of the first cycle and at the end of the fourth cycle. Groups are NN1: Normoxia after first cycle, IHH1: Hypoxia after fourth cycle; NN4: Normoxia after fourth cycle, IHH4: Hypoxia after fourth cycle. Data are expressed in mean ± SEM. Significant differences (* *p* ≤ 0.05) vs. Normobaric normoxia.

## Supplementary Materials

The following are available online at http://www.mdpi.com/1422-0067/19/2/366/s1.
