# Supplementary material for: Mechanisms of Cardiovascular Protection Associated with Intermittent Hypobaric Hypoxia Exposure in a Rat Model: Role of Oxidative Stress
Source: Int J Mol Sci. 2018 Jan 26;19(2):366. doi: 10.3390/ijms19020366 (PMC5855588; doi:10.3390/ijms19020366)
Supplement: Supplementary file 1 [file ijms-19-00366-s001.pdf]

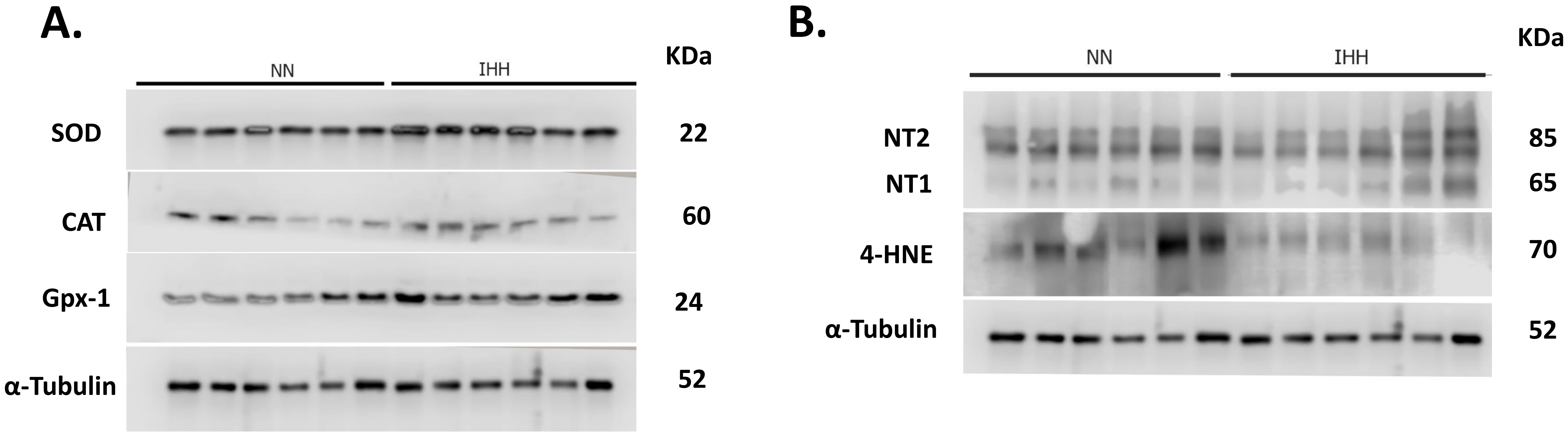

Supplementary Figure 1. Representative figures of Western Blots for the antioxidant enzymes superoxide dismutase (SOD), glutathione peroxidase (GPx) and catalase (CAT) (A), and for the oxidative stress markers nitrotyrosine-1 (NT1), nitrotyrosine-2(NT2), and 4 Hydroxynonenal (4 HNE) (B).
